# 

**Published:** 1917-03-31

**Authors:** 


### Medical Appointments.

Dr. T. W. Wade has been appointed pathologist and bacteriologist under the Monmouthshire county scheme for the treatment of venereal disease, at a salary of £550 per annum.

Miss Amelia Benner, M.B., B.Ch., the assistant schools medical officer, has been appointed to the medical charge of the maternity and child-welfare scheme at Newport (Mon).

Mrs. Edith Shannon, whose husband is serving with the Forces in the East, has been appointed resident assistant medical officer of the Sick Home of the West Ham Board of Guardians. The appointment is for the period of the war, and the salary is £300 a year.

Mr. L. J. Pisani, F.R.C.S. (Eng.), has been appointed by the Metropolitan Asylums Board as consultant in the treatment of ophthalmic cases in the Board's asylums at an inclusive fee of £5 5s. per visit, the maximum number of his visits during the year to be twenty.

### RUSSIAN ORDER FOR ENGLISHWOMEN.

In recognition of her efforts in raising funds for the provision of a Chester bed in the Anglo-Russian Hospital at Petrograd, the Mayoress of Chester (Mrs. J. M. Frost) has been presented with the Empress of Russia's special Red Cross Order. A similar honour has been conferred on Miss Craven, of Gainsborough, through whose instrumentality a Gainsborough bed is being maintained at the Petrograd Hospital

### WHAT ONE SECRETARY IS THINKING

**We publish each paragraph practically as received, without comment. Contributions to this Column and discussion on the points raised will be welcomed.)**

### Is it Expedient?

When a member of a hospital board of management is also a member of the board of a second and entirely separate institution, there are advantages as well as distinct disadvantages. The advantages consist in the unquestioned fact that by comparison of the two methods followed in the departments of the secretary, the matron, and the steward, the mind of the member concerned is easily able to judge which are, in his opinion, the better, and to give his colleagues on the two different boards his reasons for thinking so. He can tell in a moment (or he should be able to do so) which of the two secretaries conducts the better money-raising ways; he can tell which of the two matrons maintains the better discipline among the nursing staff; he can tell which of the two stewards is the better provider and manager of table commodities. But there are weighty disadvantages in the position of serving on two boards. The most serious is this: supposing that one board hits upon a novel scheme for enlarging its "public," a scheme that is new and original and the success of which depends upon its secret working for a few months, or even longer. What might happen? Would the member of the board be satisfied to see one of "his" two hospitals thrive much better than the other one, or would he pass on to the second hospital the promising information gained at the meeting held at the first hospital? We have in mind a certain worthy baronet who is a member of the board of a particularly prosperous institution and who is also a member of an equally deserving but always-in-debt institution. In this instance it seems only fair to conclude that the baronet is very conscientious, and that he strictly keeps from one group of colleagues "secrets" he hears from the other group. Still, the position might prove, to say the least, a difficult and delicate one.

Rates of Subscription: Twelve months, 6/6; Six months, 4/-; Three months, 2/-; post free to any address in the United Kingdom. Abroad, Twelve months, 8/8; Six months, 5/-; Three months, 2/6, post free. The Hospital "Index" to Volume LX., April to September 1916, is now ready, price 2½d. per copy, post free. Address The Manager, "The Hospital," The Hospital Building, 28/29 Southampton Street, Strand, London, W.C. 2.

